# What Symbionts Teach us about Modularity

**DOI:** 10.3389/fbioe.2013.00014

**Published:** 2013-11-04

**Authors:** Manuel Porcar, Amparo Latorre, Andrés Moya

**Affiliations:** ^1^Cavanilles Institute of Biodiversity and Evolutionary Biology, Universitat de València, València, Spain; ^2^Fundació General de la Universitat de València, València, Spain; ^3^Unidad Mixta de Investigación en Genómica y Salud, Fundación para el Fomento de la Investigación Sanitaria y Biomédica de la Comunitat Valenciana – Salud Pública, València, Spain

**Keywords:** modularity, orthogonality, symbiosis, endosymbionts, systems biology

## Abstract

The main goal of Synthetic Biology (SB) is to apply engineering principles to biotechnology in order to make life easier to engineer. These engineering principles include modularity: decoupling of complex systems into smaller, orthogonal sub-systems that can be used in a range of different applications. The successful use of modules in engineering is expected to be reproduced in synthetic biological systems. But the difficulties experienced up to date with SB approaches question the short-term feasibility of designing life. Considering the “engineerable” nature of life, here we discuss the existence of modularity in natural living systems, particularly in symbiotic interactions, and compare the behavior of such systems, with those of engineered modules. We conclude that not only is modularity present but it is also common among living structures, and that symbioses are a new example of module-like sub-systems having high similarity with modularly designed ones. However, we also detect and stress fundamental differences between man-made and biological modules. Both similarities and differences should be taken into account in order to adapt SB design to biological laws.

## Introduction

Beyond standardization and abstraction, modularity is one of the engineering pillars of Synthetic Biology (SB). Modularity is the degree to which components of a system can be separated and recombined. In industrial design, modularity refers to the technique that allows building larger systems by combining smaller sub-systems. In biological sciences, the term is often used to simply design the existence of “functional blocks” in organisms, but the exact meaning can vary depending on the discipline or even authors. For example, modularity might refer to a quantitative value, a proportion of the within/between modules connection ratio (Tamames et al., 2007); or as in Ecology, to the absence of interactions between modules (Galis et al., 2001). The fact that biological modules are expected to be relatively independent, is related with orthogonality, another common buzzword in SB. Orthogonality is a term broadly generalized in engineering and borrowed from mathematics, where it refers to the independence of behavior (de Lorenzo, 2011). In geometry, for example, two Euclidean vectors are orthogonal if they are perpendicular (orthogonal in fact means “straight angle”). In SB, orthogonality has been defined as the degree to which parts derived from a parent part can be tuned to the point of non-interference while maintaining the same basic conceptual function (Lucks et al., 2008). Biological parts used in life engineering should be orthogonal (independent) from each other and from the host chassis (Cheng and Lu, 2012). Modules are combinations of parts that are also expected to be independent from each other since, in engineering, modules are designed for a specific task and also designed not to alter those of other modules. The main goal of SB is plainly to make life easier to engineer and, in order to do so, life engineers combine modules to create complexity (Endy, 2005). In this work, we scrutinize whether modularity occurring in living systems, particularly in symbionts, coincides with the conception adopted by synthetic biologists. As we will show, natural and artificial systems are organized in a hierarchic fashion based on modular or module-like sub-systems. Nevertheless, a close analysis of both systems reveals similarities as well as fundamental, enlightening differences.

## Modularity in SB

In SB, DNA sequences such as genes, promoters, or terminators are generically considered as biological “parts”: building blocks from which more complex systems can be created. In order to be able to construct biological systems, a common understanding of the functioning of these parts is needed, and they should be well characterized and readily available (Müller and Arndt, 2012). The best-known biological parts are BioBricks™, named after the metaphor of biological building blocks, which form the basis of the international Genetically Engineered Machine (iGEM) competition. Students attending this competition have at their disposal the catalog of the registry of biological parts, a bank with thousands of DNA parts with a variable degree of characterization. BioBricks™ can be chosen from the registry and then combined into simple combinations such as promoter-ORFs, or into relatively complex constructs, such as oscillatory circuits or logical gates mimicking those of Electronics (Goodman, 2008). The level of complexity combining a few biological parts, which interact to yield a given useful function or behavior, constitutes a “device.” Devices, in turn, can be combined in more complex structures. For example, an engineered bacterium with the ability to “sense” pollutants and to degrade them might have detection, reporting, and catabolic networks, which are designed for specific purposes and only interact with each other at the interface level, and in a controlled and predictable fashion. This complexity level is that of “circuits” or even “systems”: holistic behaviors arising from the combined behavior of different devices. All these complexity levels, particularly those of the devices and circuits, are supposed to exhibit an orthogonal and thus modular behavior. This hierarchical organization is a consequence of the engineering inspiration of SB and it also reflects the rational design that is inherent to the engineering view of SB (Delgado and Porcar, 2013; Gramelsberger, 2013). A question that arises is whether the hierarchical organization found in natural biological systems is similar to the one described in SB, and eventually the commonalities between natural evolving systems, without design, and synthetic ones. More importantly, are the biological networks in wild-type living forms organized as the modules described in SB?

## Modularity in Natural Systems: Plasmids

During evolution, organisms have often experienced dramatic genetic exchanges, or “genetic grafts,” among members of the three main domains of life. These events, unlike classical evolution through nucleotide changes in extant genes, imply the introduction of foreign genes, plasmids, or even whole organisms into a host cell or individual, which can confer brand new functions to the new “grafted” organism, as well as open up new ecological niches, including access to trophic resources or antibiotic resistance (Wiedenbeck and Cohan, 2011). Function gain is associated to mechanisms involving gene exchange such as horizontal gene transfer (acquisition of DNA from a different species), hybridization (sexual inter-specific reproduction), or endosymbiosis (when a bacterium lives inside another organism). The combined action of these mechanisms makes the “tree of life” metaphor inaccurate. Indeed, a web might be a more precise image (Olendzenski and Gogarten, 2009). Horizontal gene transfer is often mediated by plasmids, extra-chromosomal mobile elements as small as 1 kb or as large as 1000 kb, three times larger than the smallest bacterial chromosome reported to date (López-Madrigal et al., 2011; McCutcheon and von Dohlen, 2011). Naturally occurring plasmids often encode for catabolism genes, pathogenicity, or virulence factors, and their gain or loss implies the gain or loss of such abilities. An extreme example of this plasmid-dependent phenotype is the *Bacillus cereus* group, which includes *B. cereus sensu strictu*, a human opportunistic pathogen, *Bacillus thuringiensis*, an insect pathogen used as biopesticide and as a source of genes for the construction of transgenic plants, and *B. anthracis*, the feared producer of anthrax disease (Rasko et al., 2005). These different ecological functions are linked to plasmids. In fact, if a *B. thuringiensis* strain loses its parasporal crystal forming ability through the loss of a plasmid, it becomes virtually indistinguishable from *B. cereus* and can be classified as such (Minnich and Aronson, 1984). Similarly, anthrax in chimpanzees has been linked to a *B. cereus*-like strain with *B. anthracis* virulence plasmids (Klee et al., 2010). These reports reveal that, although orthogonality is a relative concept here because plasmids cannot coexist if they have incompatible replication origins, plasmids do, and in fact, they play a key role as specific ecological behavior determinants.

It is not by accident that plasmids were chosen early in the Molecular Biology revolution as vectors to transform a range of prokaryotic and eukaryotic cells. The ease of transfer of bacterial plasmids, their linkage to precise key functions, and their independence from the chromosomal gene pool demonstrate that such mobile genetic elements can be considered as the closest biological version of engineered modules, and somehow a natural version of SB *avant la lettre*. But plasmids are not the only module-like natural genetic structure. From smallest to largest, modular-like structures exhibiting modular features (a tight link to a phenotype or function, exchangeable nature and a certain level of orthogonality) are: genes, operons, gene clusters, pathogenicity islands, plasmids, and genomes. They all confer functions, and travel around the biosphere from one host to the other with relative stability. This list is evidence of a clearly hierarchical structure: genomes might encompass plasmids, they can both bear pathogenicity islands or gene clusters with or without operons, and genes are the basic biological parts from which all this complexity is organized. In the case of genomes, which might even be smaller than large plasmids, as mentioned above, they can be seen as module-like structures of a larger entity in the case of symbiosis.

## Symbiosis

Symbiosis constitutes an alternative mode of genetic inheritance and provides abrupt, selectable genetic variation for natural selection (Gilbert et al., 2012). Symbiosis is universal and affects all branches of the tree of life (Moya et al., 2008). It ranges from the well-balanced association between fungi and algae, to yield lichens, to microbial communities in human or termite guts, with mutualistic commensalistic and strict mutualistic roles as well as intracellular endosymbiosis. In the present work, we mainly discuss mutualistic symbioses as examples of module-like natural living systems.

As already stated, synthetic systems in modular design are built by dividing the system into smaller sub-systems that are independently created and are later integrated into a whole with new functions. This is exactly the way symbionts like lichens originated, except for the fact that design, *per se*, is absent in natural history. Fungi and algae in lichens, coral polyps and zooxanthellae in coral reefs or cows, and their cellulolytic rumen bacteria, all share many of the modular design requirements: they are made of discrete self-contained functional sub-systems and they have well-defined modular interfaces. A third requirement, their standard nature, is less obvious. At first sight, there is no such a thing as a “rumen chassis” or a “standard cellulolytic bacterium” or “standard photosynthetic module.”

All module features, including the hardest to find in natural biological systems such as standardization, are present in a particular symbiotic-like extreme process known as kleptoplasty, a phenomenon in which plastids are sequestered from ingested algae by a host, which uses them for photosynthesis. The best-known case is that of *Elysia chlorotica*, a “solar-powered” sea gastropod, with an intense green color due to the massive presence in its body of chloroplasts of the marine heterokont alga *Vaucheria litorea*. Interestingly, the same chloroplasts work similarly in two radically different environments: an alga and a gastropod, and this is achieved without gene transfer between them (Bhattacharya et al., 2013). This is thus a good example of standardization of the modules in the natural world.

## Endosymbiosis, Modularity, and Metabolic Complementation

A particular case of mutualism is endosymbiosis, in which one member of the consortium lives inside the other. At present, the two paradigmatic endosymbiosis are those that took place between prokaryotes and primitive eukaryotes, which ended up in the two canonical eukaryotic organelles: mitochondria and chloroplasts. It is difficult to imagine a natural structure closer to a module than eukaryotic organelles and bacterial endosymbionts. They display clear physical, genetic, and functional limits and are specialized to perform one of several tasks essential for the host. The original bacterial genomes (alpha-proteobacteria and cyanobacteria for mitochondria and chloroplast ancestor, respectively) have reduced drastically, with a portion of the protein-encoded genes being transferred to the eukaryotic nuclear genome. Other genes have simply been lost, and their function replaced by the host (Latorre et al., 2011).

Symbiotic associations between prokaryotes and uni- and multi-cellular eukaryotes seem to be present in every major branch of the tree of life (Moya et al., 2008). Similar to what happened in eukaryotic organelles, genome reduction is a common feature of bacterial endosymbionts in the process of adaptation to their multicellular eukaryotic hosts. An inevitable consequence of the massive loss of genetic material is the loss of modularity as compared to modularity in free-living relatives. In this context, a module is defined as a part of a network with abundant connections between the nodes within it, and less connected to nodes outside the module (Tamames et al., 2007). Interestingly, this notion is similar to the idea of “relative orthogonality” in SB (de Lorenzo, 2011). Modularity values can be calculated as the ratio intra-module/inter-module connections. With this simple method it has been observed that in protein-protein interaction networks, *Buchnera aphidicola* BAp, primary endosymbiont of the aphid *Acyrthosiphon pisum*, loses modularity with respect to *E. coli* (Tamames et al., 2007) because the connections inside modules are particularly affected by the reduction process compared to connections among modules. Many of the proteins inside the three big modules of the *E. coli* network corresponding to cell division and chaperones, RNA polymerase and DNA metabolism are lost in *Buchnera*, which concomitantly leads to a loss of modularity, although the most connected proteins between modules (hub connectors) are preserved.

A question that arises is what happens with the loss of essential bacterial functions that cannot be carried out by the eukaryotic host. One solution is that some of them are transferred to the host, similar to eukaryotic organelles. However, it does not seem to be the case in endosymbionts with reduced genomes (Husnik et al., 2013). Rather, the solution seems to lie in the appearance of metabolic complementation, a convergent solution adopted in several cases by endosymbionts with small genomes living in consortium with other endosymbionts. One example is *B. aphidicola* BCc, primary endosymbiont of the aphid *Cinara cedri*, which has the smallest genome among all the sequenced *Buchnera* strains. *B. aphidicola* BCc has established metabolic complementation with the secondary endosymbiont *Serratia symbiotica* SCc for the provision of various metabolites necessary for both the host and themselves. In general, *B. aphidicola* BCc synthesizes essential amino acids whereas *S. symbiotica* SCc is in charge of vitamin provision. A similar situation is found in the symbiotic systems of the sharpshooter *Homalodisca coagulata* and in the cicada *Diceroprocta semicincta*. Both insects harbor a Bacteroidetes endosymbiont, *Sulcia muelleri*, which needs to be complemented by a second endosymbiont (*Baumannia cicadellinicola* and *Hodgkinia cicadicola*, respectively) to fulfill its symbiotic role. *S. muelleri* synthesizes most of the essential amino acids, whereas the second symbiont synthesizes vitamins. These cases of metabolic complementation are compatible with the notions of modularity and orthogonality according to SB terminology. If we consider the synthesis of amino acids and vitamins as being performed by two independent modules for the synthesis of amino acids and vitamins, respectively, then the loss of some devices in any given endosymbiont will be complemented by the other one, thereby maintaining the function of both modules in the whole system. From the functional point of view, modularity is maintained in the form of a hybrid module incorporating both members of the consortium.

An extreme case of metabolic complementation is found in the nested endosymbiosis of mealybugs of the subfamily Pseudococaine, such as *Planococcus citri*, where and endosymbiont *Moranella endobia* is located inside *Tremblaya princeps*, harboring the smallest genome reported so far (López-Madrigal et al., 2011; Husnik et al., 2013). In this case, the complementation involves not only metabolic but also informational functions as *T. princeps* appears to be a mere factory for amino acid synthesis and translating proteins, using the precursors provided by *M. endobia* including some informational proteins. The loss of modules in the case of *T. princeps* is massive, whereas *M. endobia* behaves as other previously reported cases, like that of *B. aphidicola* BAp.

Finally, a striking case of metabolic complementation is the synthesis of tryptophan in *C. cedri*. In this case, *B. aphidicola* BCc has preserved the first two genes of the pathway, coding for anthranilate synthase, whereas the rest of the genes are located in the *Serratia* chromosome (Gosalbes et al., 2010; Lamelas et al., 2011). If the tryptophan operon is considered as a device within the module for synthesis of essential amino acids, in this consortium the device is composed of parts from two genomes, emerging as a new device to perform the same function. Not unlike the BioBricks™ used in the iGEM competition, parts of different origin are combined – by evolution in the natural system, instead of rational design – into a genetically hybrid device.

It is difficult to imagine a natural structure closer to a module than eukaryotic organelles and bacterial endosymbionts. They display clear physical, genetic, and functional limits; they are specialized to perform one of several tasks that are imperative for the host; and they are mobile. Mobility, which directly relates to standardization, as only standard sub-systems work in different systems, has been proven by historical endosymbiotic events, both ancient and recent.

## Perspectives and Conclusion

Modularity is evident in the way living beings are organized. The hierarchical gene-to-genome structure of cells has many intermediate structures that share most of the features of engineered modules. Plasmids and endosymbionts are the closest biological representatives of modules, and they both share clear limits, specialized functions, ease of transfer, and a relatively high extent of orthogonality or independence of behavior, as well as standardization or multiplicity of contexts in which they can work. The main difference between life and machines is the way their complexity arises. In contrast with rationally designed artificial systems, life is a consequence of natural selection, and evolution has resulted in entangled genetic, metabolic, and symbiotic networks, which are as functional as rationally designed ones, but much more recalcitrant to rational modification. Further development of Systems Biology and modeling, and flexibility in the application of engineering tenets to biotechnology might be key factors for a successful symbiosis-inspired refactoring of living forms.

It is also highly relevant to develop the rationality behind the role of bacterial species as modules in complex communities dealing with eukaryotes such as those present in the human gut, and exploring the concept of physiological alternative multi-species modules. Interestingly, there is general assent on using synthetic microbial consortia as one of the new frontiers of SB (Shong et al., 2012). Likewise, the potential of engineering sub-populations of bacterial cells, each contributing to a global behavior, has recently been stressed (Bacchus and Fussenegger, 2013). As a final conclusion, our current knowledge on biological systems reveals the existence of robust compartments, ranging from plasmids or metabolic complementation genes to endosymbionts, many of which share most of the requirements set by SB for orthogonal modules, although orthogonality or independent behavior is almost always limited in biological modules. Further studies of the complexity of microbiomes, endosymbiosis, and minimal cells and the development of new selection-based tools in SB (Porcar et al., 2011) may be very helpful to pave the way toward the construction of truly modular synthetic living systems.

## Conflict of Interest Statement

The authors declare that the research was conducted in the absence of any commercial or financial relationships that could be construed as a potential conflict of interest.
